# miRNA-182 regulated MTSS1 inhibits proliferation and invasion in Glioma Cells

**DOI:** 10.7150/jca.47588

**Published:** 2020-08-01

**Authors:** Zhexuan Li, Longbo Zhang, Zhiqiang Liu, Tianxiang Huang, Ying Wang, Yujie Ma, Xingqi Fang, Yanqing He, Yangying Zhou, Lei Huo, Jun Wu

**Affiliations:** 1 Department of Neurosurgery, Xiangya Hospital, Central South University, Changsha, 410008, Hunan, China.; 2 Department of Oncology, Xiangya Hospital, Central South University, Changsha, 410008, Hunan, China.

**Keywords:** Gliomas, MTSS1, miR-182, EMT, Proliferation, Invasion

## Abstract

Human glioma is the most common malignant and fatal primary tumor in the central nervous system. Currently, the high incidence and low cure rate of glioma make it a considerable threat to human health. Thus, elucidating the molecular mechanisms of glioma development and progression has become a major focus to identify new and effective biomarkers and improve the comprehensive neurosurgical treatment of glioma from the basic research and clinical perspectives. In our present study, we aimed to investigate the expression pattern and biological function of Metastasis suppressor protein 1(MTSS1) in glioma and to further explore whether miRNAs were involved in the deregulation of MTSS1. By overexpressing MTSS1 in highly malignant human glioma cells, we discovered a role for MTSS1 in suppressing the proliferation and invasion of glioma cells, and we showed that MTSS1 participated in transforming growth factor-beta 1 (TGF‐β1) -induced epithelial‐mesenchymal transition (EMT) in glioma cells. Biochemical analyses suggested that miR-182 may target MTSS1 and that miR-182 expression is negatively correlated with MTSS1 expression in glioma tissues. This finding was further confirmed by luciferase reporter experiments. Furthermore, a miR-182 inhibitor induced glioma cell proliferation and invasion by increasing MTSS1 expression. In conclusion, we believed that miR-182 modulates glioma cell migration and invasion by targeting the MTSS1 and suggested that miR-182 was a potential therapeutic target for gliomas.

## Introduction

Gliomas are the most common malignant primary intracranial tumors, accounting for 46% of intracranial tumors 1-4. Gliomas generally show infiltrative growth and glioma cells can be found in normal brain tissues with up to 2 cm outside the tumor focus 5. Even if the tumor is completely removed by clinical surgery, it may inevitably recur at the edge of the surgical cavity 6. Therefore, it is difficult to completely remove glioma cells during surgery. However, the therapeutic effect of radiotherapy and chemotherapy on glioma cells is not obvious. Therefore, clarifying the pathogenesis of gliomas and identifying new effective therapeutic targets are challenging and becoming popular topics in neurosurgery.

MTSS1 is a metastasis suppressor in several cancers. Recently, the role of the MTSS1 in tumor development, especially in invasion and metastasis, has been reported. MTSS1 is also called missing in metastasis (MIM), MIM-B, BEG4 and LIAA042. MTSS1 was first identified in human bladder cancer cells 7. MTSS1 is expressed in most normal tissues and a few nonmetastatic cancer cell types, but its expression is significantly reduced or even absent in many metastatic tumors. For example, in metastatic prostate cancer 8, gastric cancer 9 and renal cancer 10, MTSS1 may play an antimetastatic role in tumor progression. Overexpression of MTSS1 can significantly inhibit the invasion, migration and growth abilities of breast cancer cells 11. More studies on the transcriptional or posttranscriptional regulation of MTSS1 and the regulation of MTSS1 at the protein level are needed.

EMT is an important process promoting cancer proliferation and invasion. EMT is characterized by loss of cell polarity and adhesion and increased invasion and migration 12. Tumor cells can produce several growth factors to induce EMT, among which TGF‐β1 is one of the most important factors. TGF‐β1 induces the transformation of cancer cells from a highly differentiated morphology to a migratory phenotype 13.

MicroRNAs (miRNAs) are short RNA molecules with a length of 19 to 25 nucleotides. A single miRNA can influence the expression of many genes and target hundreds of mRNAs. MiRNAs are often involved in functionally interactive pathways and playing roles in RNA silencing and posttranscriptional regulation of gene expression 14-17. In addition, miRNAs control cellular processes such as proliferation, growth, death, inflammation, and development 18. As oncogenes or tumor suppressors, miRNAs are involved in the development and progression of cancers. For example, miR-182 promotes prostate cancer progression by activating the Wnt/βcatenin signaling pathway 19, and miR-182 enhances radio-resistance in non-small cell lung cancer cells by regulating FOXO_3_ 20. However, whether miR-182 can directly target MTSS1 and affect the progression of glioma is unclear.

Considering the possible role of MTSS1 in tumorigenesis and development, this study preliminarily investigated the expression and biological function of MTSS1 and the mechanism regulating its expression in human gliomas.

## Materials and Methods

### Patients and samples

Thirty-four human glioma and matched normal tissues were obtained from patients who underwent surgery between March 2013 and March 2014 in the Department of Neurosurgery in Xiangya Hospital, Central South University, Changsha, Hunan, China. All patients received informed consent before surgery, and all experiments were conducted in accordance with the bioethical standards issued by the Research Ethics Committee of Central South University. The main clinical and pathological parameters of the glioma patients are summarized in Table 1.

### Cell culture and transfection

The malignant human glioma cell lines U87MG and T98G were purchased from the Cell Center of Central South University, Changsha, China. The cells were cultured in DMEM (Dulbecco's modified Eagle's medium) (30023.01; Cytiva, MA, USA) supplemented with 10% fetal bovine serum (16000044; ThermoFisher, MA, USA) in an atmosphere of 37°C and 5% CO_2_. All cell lines were tested to ensure that they were free of contamination, and cells in exponential growth phase were used for subsequent experiments. Glioma cells were transfected with the miR-182 inhibitor/inhibitor NC (negative control) (GenePharma, Shanghai, China) according to the manufacturer's protocol 21.

### RNA extraction, reverse transcription and qRT-PCR

Total RNA was extracted from tissues or cells using TRIzol reagent (Invitrogen, Carlsbad, CA, USA). An All-in-One™ miRNA qRT-PCR Detection Kit (GeneCopoeia Inc, MD, USA) was applied for reverse transcription and quantitative detection of miRNAs according to the user manual. MiRNA qRT-PCR primers were used to measure the expression of miRNAs, and GAPDH was used as the control for PCR. Finally, miRNA levels were quantified using the 2-ΔΔCT method. The sequences of the primers used for PCR are listed in Table S1.

### Immunohistochemistry

The tissue chips were baked in an oven at 60 °C for 2-4 hours, deparaffinized in xylene twice for 10 min each, and rehydrated sequentially in anhydrous ethanol for 10 min, 95% ethanol for 10 min, 75% ethanol for 10 min, 50% ethanol for 10 min, and ddH2O for 10 min. Antigen retrieval was conducted in 10 mmol/L citric acid buffer (pH 6.0) in a microwave oven at 100 °C for 15 min. Endogenous peroxidase activity was blocked with 3% hydrogen peroxide for 10 min at room temperature. Slides were incubated overnight at 4 °C with an anti-MTSS1 mouse polyclonal antibody (SC101204; 1:1000, Santa Cruz Biotechnology, TX, USA) and an HRP-labeled goat anti-mouse polymer (HS20101; 1:3000 dilution, TransGen Biotech, Beijing, China) was then added. Immunoreactive proteins were stained with 3',3'-diaminobenzidine, and slides were then restained with Meyer's hematoxylin. The negative control slides were reacted with normal sheep serum under the same experimental conditions.

### MTSS1 transfection

The pLEX-control, pLEX-MTSS1, and pMIR-REPORTTM miRNA expression reporter vectors were provided by the Cancer Research Institute of Central South University. Plasmids (4 micrograms of plasmid dissolved in 250 µl of Opti-MEM) were transfected into U87MG and T98G cells with Lipofectamine 2000. Forty-eight hours after transfection, 5 µg/ml puromycin dihydrochloride was used to screen stable cell lines expressing MTSS1 shRNAs for 2 weeks. Transfected cells were expanded and cultured in 3 µg/ml puromycin dihydrochloride, and the expression of MTSS1 was confirmed by Western blot analysis 22.

### CCK-8 assay

All types of glioma cells were seeded in a 96-well plate at a concentration of 2000 cells per well. The cells were then maintained at 37 °C for 24, 48, 72 and 96 hours after transfection. The cells were treated with CCK-8 (Beyotime, Shanghai, China) reagent (5 mg/ml) for 1 hour at 37 °C. The absorbance at 450 nm was measured in a microplate reader. The values from triplicate experiments were averaged 23.

### Transwell invasion assay

Transwell chambers were coated with 200ug/ml of Matrigel (356255; Corning, NY, USA) and used polycarbonate filter. Transfeted cells were seeded in Transwell cell culture inserts at a concentration of 2 × 104 cells/well. The cells were cultured at 37 °C in a 5% CO2 incubator for 48 hours. Then, the supernatants were aspirated and washed 3 times with PBS. After the cells on the Transwell membranes were wiped away, the remaining cells were fixed with 0.1% crystal violet and stained with 4% methanol. Finally, the number of cells was counted under a light microscope. The experiment was repeated 3 times.

### Luciferase reporter assays

The sequence of the MTSS1 3'-UTR (3'-untranslated region) was synthesized by chemical synthesis and fused to the luciferase enzyme sequence in the pMIR-REPORT vector. After the ligation products were transformed into competent bacteria and incubated overnight at 37 °C with shaking at 250 rpm, the plasmids were extracted. U87MG and T98G cells were seeded in a 24-well plate. Each cell line was cotransfected with 0.5 µg of luciferase reporter plasmid containing the MTSS1 promoter, 100pmol of miR-182 inhibitor and 0.5μg of marine luciferase plasmid. After 48 hours of transfection, a luciferase assay system kit was used to measure luciferase activity.

### Western blot analysis

Whole-cell protein extracts were collected before Western blot analysis was performed as described previously 24, 25. Thirty micrograms of total protein were separated by 10% SDS‐PAGE for 1 hour and were then transferred to a polyvinylidene difluoride membrane for 90 min (IPFL85R; Millipore, MA, USA). The membrane was incubated with primary mouse antibodies against MTSS1 (SC-101204; 1:1000 dilution; Santa Cruz, TX, USA), E‐cadherin (SC8426; 1:1000 dilution; Santa Cruz, TX, USA), Vimentin (10366-1-AP; 1:1000 dilution, Proteintech, IL, USA), β-actin (20536-1-AP; 1:1000 dilution, Proteintech, IL, UAS), and a goat antibody against HRP (HS201-01; 1:3000 dilution, TransGen Biotech, Beijing, China) at 4 °C overnight. After incubation, PBST (phosphate-buffered saline with Tween‐20; HyClone, Logan, UT) was used to wash the membrane 3 times at room temperature, and the membrane was incubated with HRP‐labeled goat anti‐rabbit IgG for 1 hour at room temperature. Each experiment was repeated in triplicate.

### Statistical analyses

All data were evaluated using IBM SPSS version 13.0 and GraphPad Prism 6. Student's t-test or one-way ANOVA (analysis of variance) was performed to assess the significance of differences between samples in data from three independent experiments. Categorical variables are expressed as absolute frequencies and percentages, and continuous data were analyzed with descriptive statistics (mean values). *P* < 0.05 was considered statistically significant. Two‐tailed tests were performed.

## Results

### MTSS1 levels are decreased in glioma tissues

**Figure 1:**
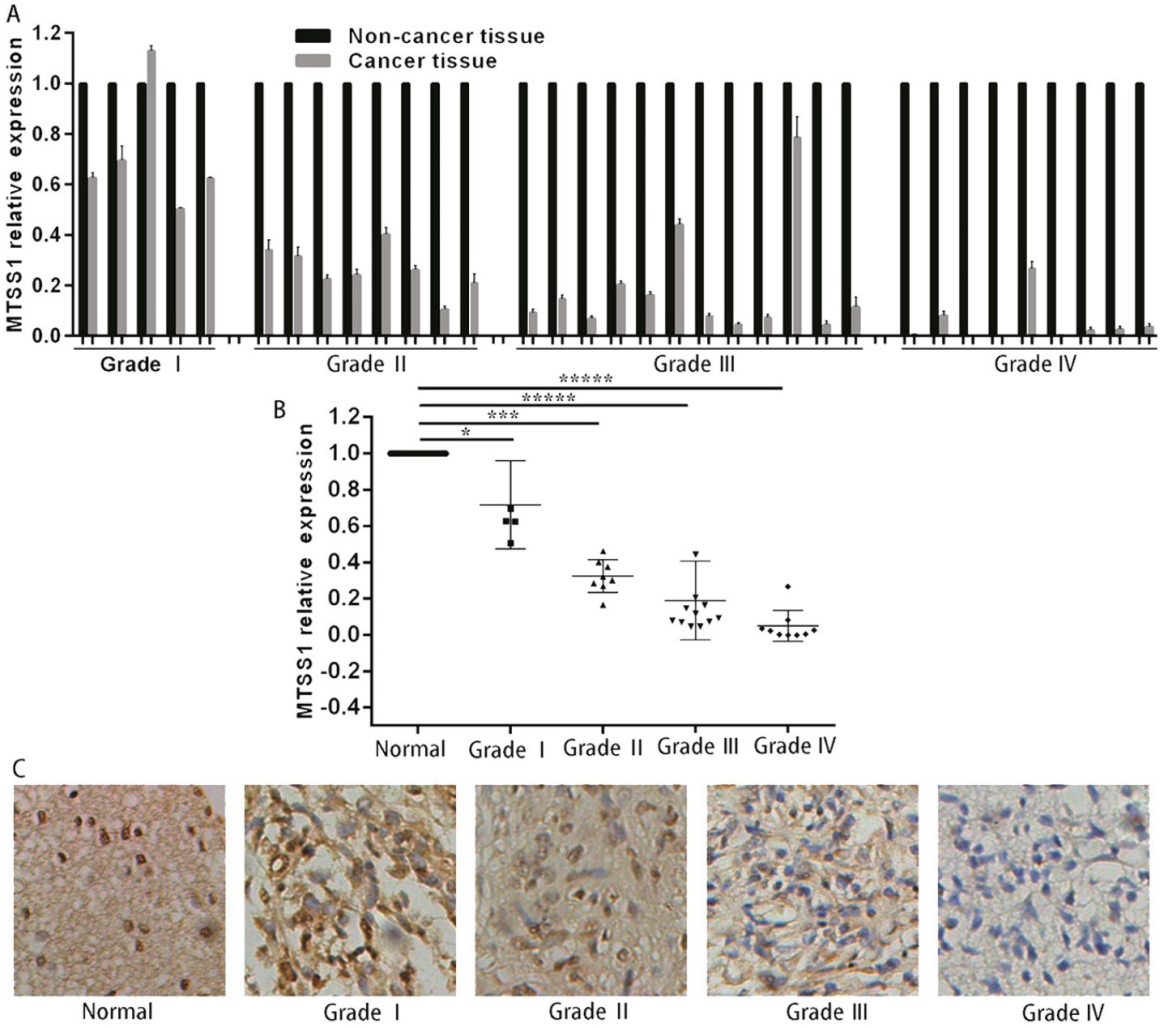
** MTSS1 levels decreased in gliomas tissues and cells**. **(A and B)** Level of MTSS1 in tissues of patients with human gliomas and their matched normal tissues at mRNA and** (C)** protein level. (*P<0.05, ***P<0.001, *****P<0.0001). MTSS1, Metastasis suppressor protein 1.

To investigate whether MTSS1 was involved in the development of human gliomas, we first detected the expression of MTSS1 in 34 human glioma and matched normal tissues, specifically, 5 grade I glioma tissues, 8 grade II glioma tissues, 12 grade III glioma tissues and 9 grade IV glioma tissues. The expression of MTSS1 in glioma tissues was significantly lower than that in the matched paracancerous tissues (Fig. 1A). The expression of MTSS1 was closely related to the grade of the gliomas—the higher the degree of malignancy, the lower was the expression of MTSS1 (Fig. 1B). To further evaluate the expression of MTSS1, immunohistochemistry was conducted in glioma tissue, specifically, 10 samples of normal brain tissue, 5 samples of grade I glioma tissue, 23 samples of grade II glioma tissue, 26 samples of grade III glioma tissue and 3 samples of grade IV glioma tissue. The protein level of MTSS1 in glioma tissue was significantly decreased compared with that in normal brain tissue. The higher the degree of malignancy of a glioma, the lower was the level of MTSS1 protein in the brain tissue (Fig. 1C). These results suggested that the expression level of MTSS1 was negatively correlated with the degree of malignancy of gliomas and may play an important role in glioma occurrence and progression.

### MTSS1 inhibits the proliferation and invasion of glioma cells

**Figure 2:**
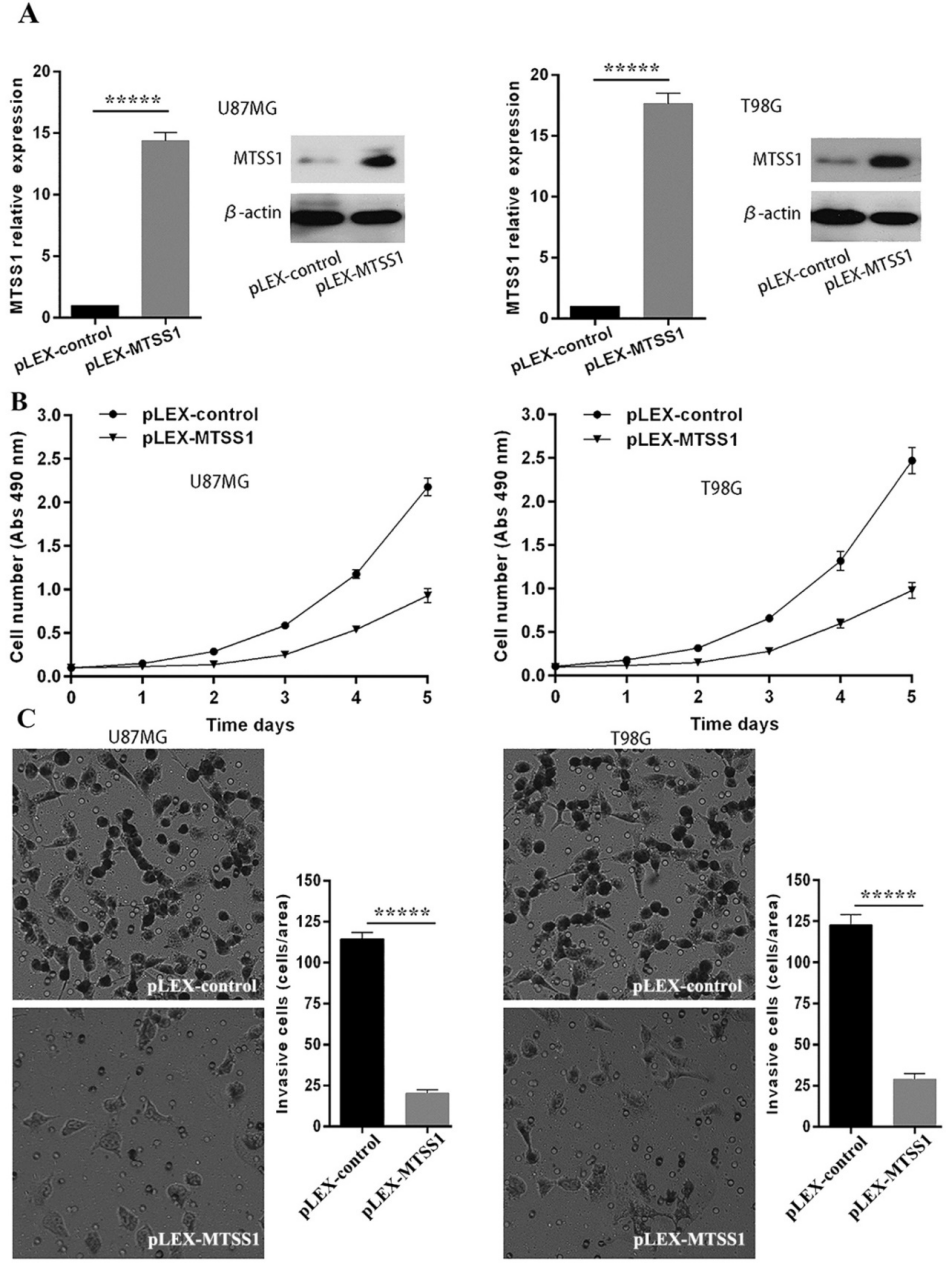
** MTSS1 inhibits the proliferation and invasion of gliomas cells. (A)** The overexpression vector of MTSS1 and the control were respectively transferred into U87MG and T98G cell lines and then tested by qPCR and Western blot analysis. **(B)** Proliferation ability was examined and quantified by CCK-8 assay. **(C)** Invasive ability was assayed and quantified by Transwell invasion assay. (*P<0.05, ***P<0.001, *****P<0.0001) MTSS1, Metastasis suppressor protein 1.

To evaluate the role of MTSS1 in glioma occurrence and progression, we selected the grade IV glioma cell lines U87MG and T98G as the research model. The MTSS1 overexpression vector and the control empty vector were separately transfected into the U87MG and T98G cell lines with a transfection reagent, and the cells were then subjected to qRT-PCR and Western blotting. Compared with that in the corresponding cells transfected with the blank control vector, the level of MTSS1 mRNA in U87MG and T98G cells was increased by 14.37- and 17.64-fold (*P* < 0.0001), respectively, after introduction of the plex-MTSS1 vector, and the level of MTSS1 protein in U87MG and T98G cells was significantly increased (Fig. 2A). Cells from each group were inoculated into 96-well plates at 1000 cells/well. Cell proliferation was evaluated with a CCK-8 assay the next day. The absorbance value represents the number of cells. Overexpression of MTSS1 significantly decreased the proliferation rate of U87MG and T98 cells (Fig. 2B). In addition, we used a Transwell assay to evaluate the invasion ability of cells. Overexpression of MTSS1 significantly decreased the invasion ability of U87MG and T98 cells, as evidenced by the significantly reduced number of cells crossing the matrix barrier (*P* < 0.0001) (Fig. 2C).

### MTSS1 participates in TGF‐β1‐induced EMT in glioma cells

**Figure 3:**
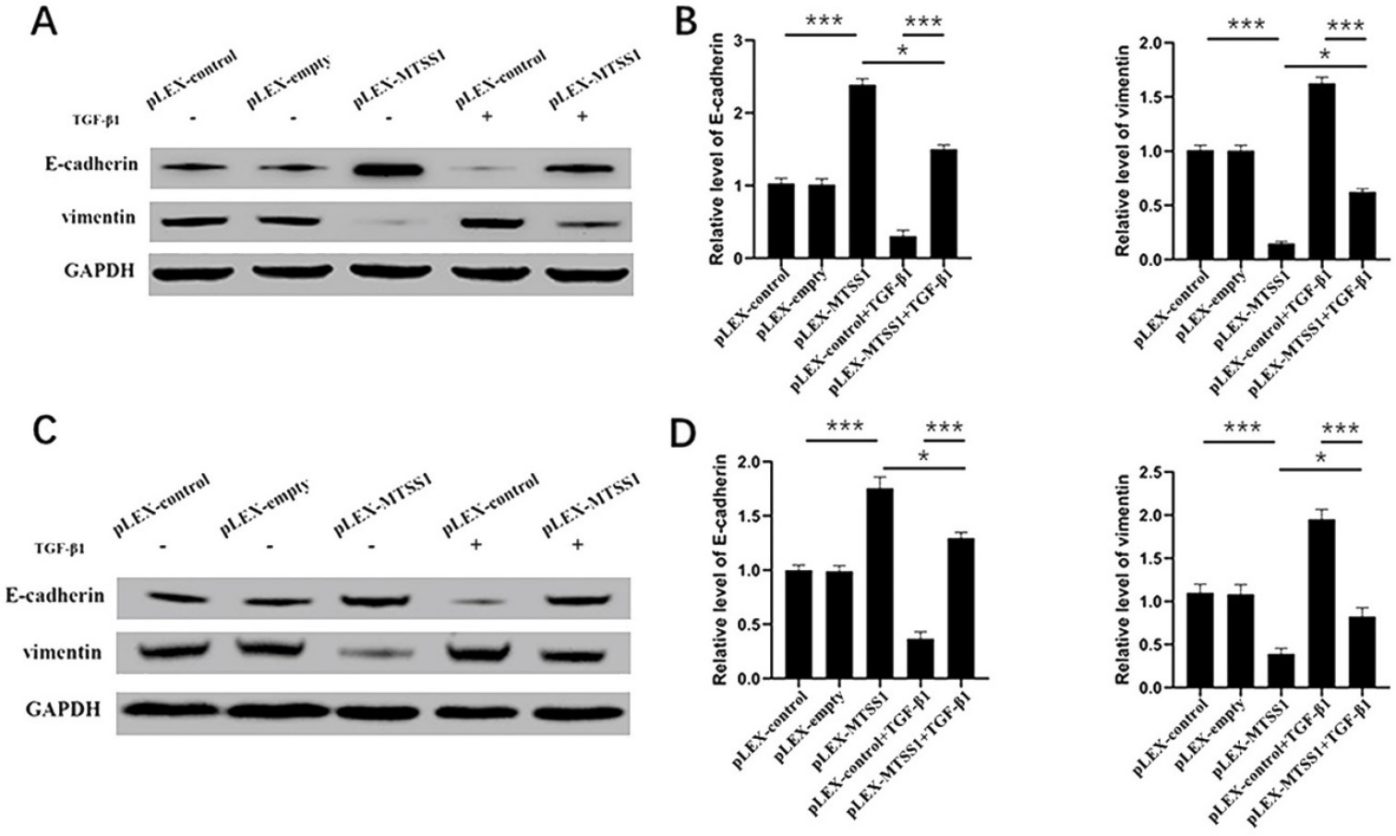
** MTSS1 participated in TGF-β1-induced EMT in gliomas cells. (A, B)** The protein levels of epithelial markers (E-cadherin) and mesenchymal markers (vimentin) in U87MG and T98G **(C, D)** cells were assessed using Western blot analysis. (*P<0.05, ***P<0.001, *****P<0.0001) MTSS1, Metastasis suppressor protein 1. EMT, epithelial-mesenchymal transition, TGF-β1, transforming growth factor beta 1.

During the process of transformation from benign tumors to malignant and metastatic tumors, the most obvious morphological change is the transformation of tumor cells from a highly differentiated epithelial morphology to a mobile and invasive phenotype 26. Therefore, EMT is considered one of the most critical steps in cancer metastasis 27. Next, we explored the impact of MTSS1 on EMT. As expected, the Western blot results indicated that overexpression of MTSS1 significantly increased E-cadherin expression and decreased vimentin expression in U87MG cells (Fig. 3A and 3B) and in T98G cells (Fig. 3C and 3D). These results indicate that MTSS1 can inhibit tumor invasion and metastasis by regulating EMT. TGF-β1 is a growth factor that induces EMT and participates in EMT processes. In U87MG cells, the expression of E-cadherin in the TGF-β1-positive group was slightly decreased compared with that in the negative group. In contrast, the expression of Vimentin in the TGF-β1-positive group was slightly increased compared with that in the negative group (Fig. 3A and 3B). We found the same result in T98G cells (Fig. 3C and 3D). MTSS1 regulated the EMT process induced by TGF-β1 to inhibit the proliferation and invasion of glioma cells.

### MiR-182 expression is negatively correlated with MTSS1 expression in glioma tissues

**Figure 4:**
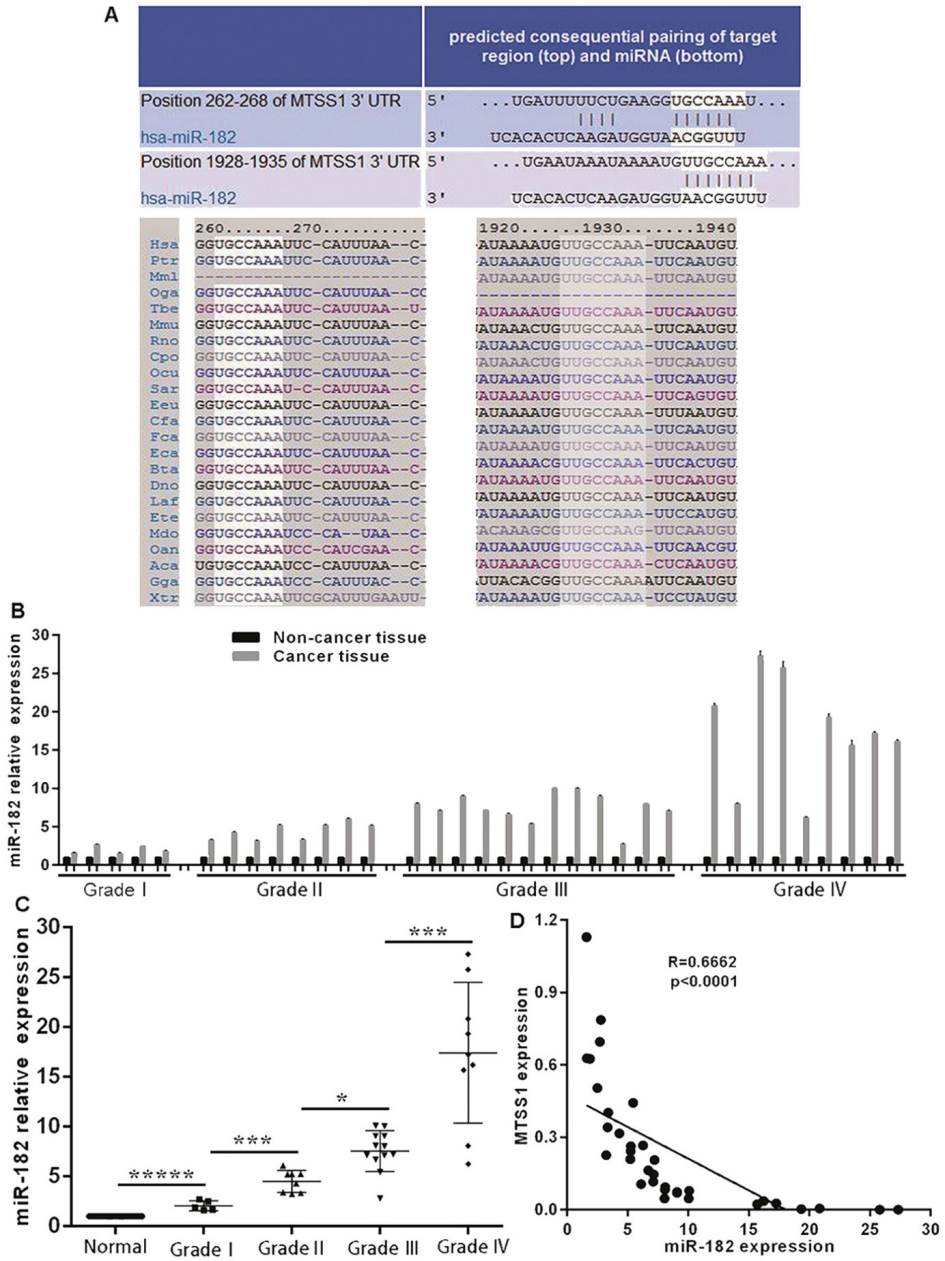
** miR-182 expression is negatively correlated with MTSS1 expression in gliomas tissues. (A)** Bioinformatics analysis show that miR-182 binding site of MTSS1 mRNA. **(B)** Level of miR-182 in tissues of patients with human gliomas at mRNA. **(C)** The expression of miR-182 was related to the grade of gliomas. **(D)** miR-182 was negatively correlated with MTSS1 in tissues of patients. (*P<0.05, ***P<0.001, *****P<0.0001). MTSS1, Metastasis suppressor protein 1.

Considering the significant downregulation of MTSS1 expression in human gliomas and its important role in the proliferation and invasion of glioma cells, it is necessary to explore the molecular mechanism underlying the downregulation of MTSS1 expression in human gliomas. To date, the role of epigenetics in tumors has received increasing attention. Here, we focused on miRNA-mediated control of gene expression and regulation. Through bioinformatics analysis, we identified a conserved miR-182 binding site at positions 262-268 and 1928-1935 in the 3'-UTR of MTSS1 mRNA (Fig. 4A). To investigate whether miR-182 was involved in the regulation of MTSS1 expression in gliomas, we first evaluated the abovementioned 34 human glioma tissues and their matched normal tissues by miRNA-specific qRT-PCR. The expression of miR-182 was significantly upregulated in glioma tissues compared with the matched paracancerous tissues (Fig. 4B), and upregulated expression of miR-182 was closely related to a higher grade of gliomas. The higher the degree of malignancy of a glioma, the higher was its expression level of miR-182 (Fig. 4C). Correlation analysis of the expression levels of miR-182 and MTSS1 in brain gliomas showed that miR-182 expression was negatively correlated with MTSS1 expression (*P* < 0.0001) (Fig. 4D), suggesting that overexpression of miR-182 might affect the regulation of MTSS1 expression.

### MiR-182 directly regulates the expression of MTSS1

**Figure 5:**
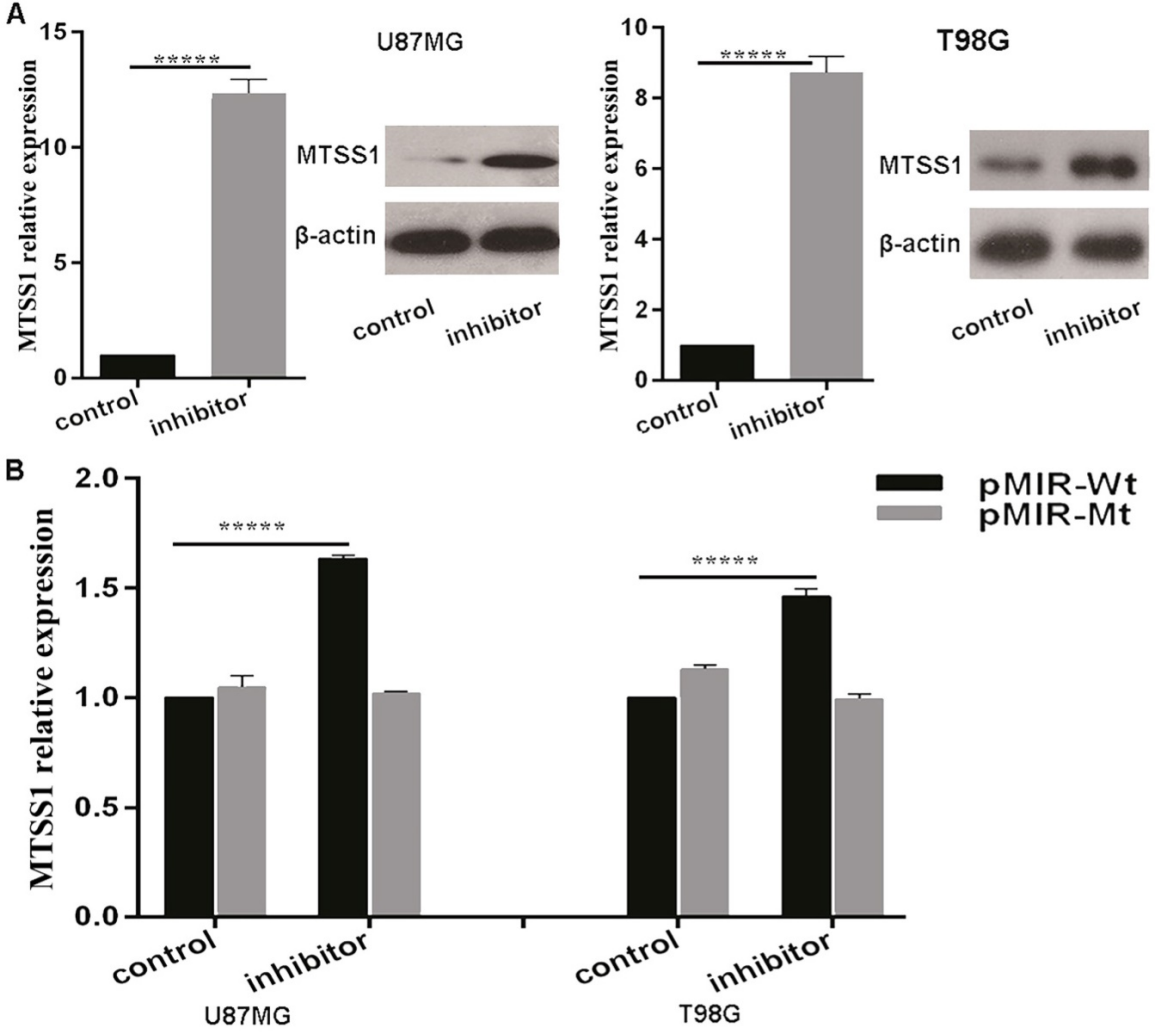
** miR-182 directly targets the expression of MTSS1. (A)** miR-182 was negatively correlated with MTSS1 at mRNA and protein levels in U87MG and T98G cells. **(B)** The luciferase activity of U87MG and T98G cells were detected miR-182 directly target MTSS1. (*P<0.05, ***P<0.001, *****P<0.0001) MTSS1, Metastasis suppressor protein 1.

The above results suggested that miR-182 may regulate the expression of MTSS1. To confirm whether miR-182 directly regulates the expression of MTSS1, we first introduced a miR-182 inhibitor and its NC inhibitor into U87MG and T98G glioma cells. qRT-PCR was used to detect the changes in the expression of MTSS1 mRNA after transfection of the miR-182 inhibitor. After treatment, the miRNA level of MTSS1 was increased 12.36-fold in U87MG cells and 8.73-fold in T98G cells. In addition, the Western blot results showed that after transfection of the miR-182 inhibitor, the protein level of MTSS1 in U87MG and T98G cells was significantly increased (P < 0.0001) (Fig. 5A). To provide direct evidence that miR-182 directly regulated the expression of MTSS1 via binding of its seed sequence to the complementary sequence in the 3'-UTR of MTSS1, we synthesized a 200-bp DNA fragment with 400 bp upstream and downstream flanking sequences of each miR-182 binding site in the 3'-UTR of MTSS1 and cloned it into the pMIR-REPORT luciferase reporter vector to construct the wild-type vector (pMIR-Wt). The DNA sequence of the 3`-UTR was cloned into the pMIR-REPORT luciferase reporter vector to construct the mutant vector (pMIR-Mt). The luciferase activity in U87MG and T98G cells were measured after cotransfection of the miR-182 inhibitor or scrambled control, pMIR-Wt or pMIR-Wtand pRL TK Renilla luciferase (a marine luciferase reporter gene vector, used as the internal control). Compared with the scrambled control, the miR-182 inhibitor increased the reporter gene activity in U87MG cells by 63.4% and increased the reporter gene activity in T98G cells by 4 5.6% (P < 0.0001) (Fig. 5B). These results indicate that miR-182 can directly affect the mRNA and protein expression of MTSS1 by complementary binding to the 3'-UTR of MTSS1.

### Downregulation of miR-182 inhibits the proliferation and invasion of glioma cells

**Figure 6:**
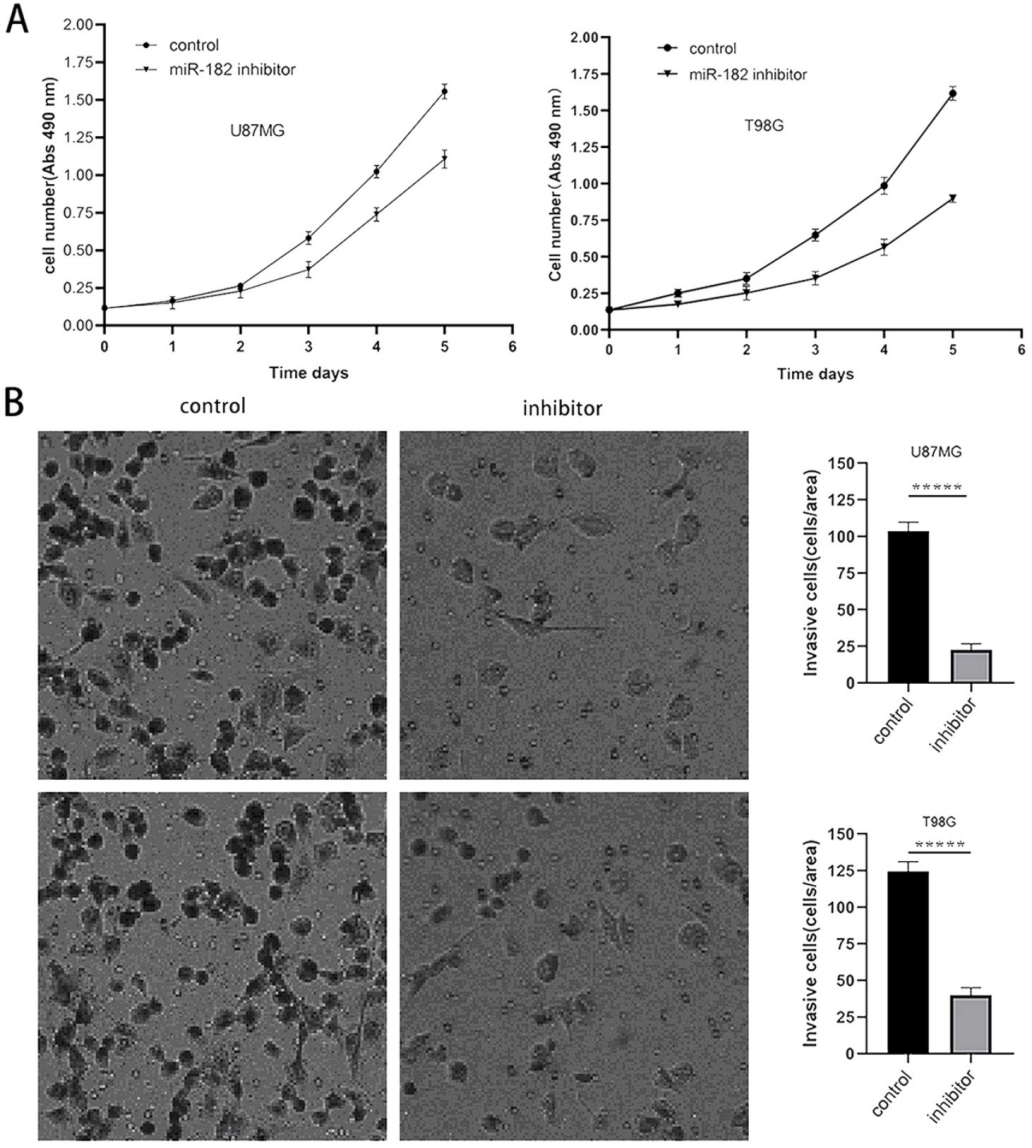
** Down regulation of miR-182 inhibits the proliferation and invasion of gliomas cells. (A)** The proliferation ability of U87MG and T98G was examined and quantified by CCK-8 assay.** (B)** The invasion ability of U87MG and T98G cells were examined and quantified by the Transwell invasion assay. (*P<0.05, ***P<0.001, *****P<0.0001) MTSS1, Metastasis suppressor protein 1.

The above experimental results showed that MTSS1 can inhibit the proliferation and invasion of glioma cells and that miR-182 can directly target and regulate the expression of MTSS1. Therefore, we sought to further clarify the biological function of miR-182 in human glioma cells. After the miR-182 inhibitor or its scrambled control was transfected into U87MG and T98G cells, the proliferation ability of these cells was evaluated by a CCK-8 assay. The proliferation ability of U87MG and T98G cells decreased significantly after the functional inhibition of miR-182 by the miR-182 inhibitor (Fig. 6A). In addition, we used a Transwell assay to evaluate the invasion ability of these cells. When the miR-182 inhibitor was used for functional inhibition of miR-182, the invasion ability of U87MG and T98G cells was significantly reduced, as evidenced by the significantly reduced number of cells crossing the matrix barrier (P < 0.0001) (Fig. 6B). These results indicate that inhibition of miR-182 and overexpression of MTSS1 had similar biological functions in human glioma cells, which strongly suggested that miR-182 may play a biological role by targeting the expression of MTSS1.

## Discussion

As we know that glioma is the most common malignant and fatal primary tumor in the central nervous system and is characterized dispersal of tumor cell 28. Here, we analyzed the molecules that play important roles in the occurrence and development of gliomas and can be used as potential markers or targets for treatment or prediction. MTSS1, as a metastasis suppressor gene, is an actin-binding protein in cytoskeleton function. This protein plays a very important role in cell-cell adhesion 29. It belongs to the IMD (IRSp53 and MIM domain) family and is a kind of actin-binding scaffold protein involved in the occurrence and metastasis of tumors 30. MTSS1 promotes actin filament assembly and is related to cytoskeletal and cellular movements by increasing RAC1-GTP expression 31, 32. Other studies have shown that MTSS1 can directly inhibit the actin nucleation of DAAM1 in dendritic protrusions. MTSS1 is epigenetically regulated and inhibits the movement of glioma cells 33. However, more research is needed on the transcriptional and posttranscriptional regulation of MTSS1.

Recently, the role of the MTSS1 in tumor development, especially in invasion and metastasis has been reported. In metastatic prostate cancer 34, gastric cancer 35 and renal cancer 36, MTSS1 may play an antimetastatic role in tumor progression. It was also literature reported that MTSS1 was regulated by epigenetics in glioma cells and inhibits glioma cell motility. Most importantly, it showed that overexpression of MTSS1 cannot alter cell apoptosis. 33. However, the molecular mechanisms of MTSS1 associated with invasion and metastasis of glioma was poorly understood.

Migration, invasion and metastasis are important processes during tumor progression. EMT is a process in which epithelial cells acquire stromal characteristics 37. Due to some physiological or pathological factors, cells lose their polarity, tight intercellular junctions and adhesive connections and acquire interstitial cell morphology and characteristics 38. In cancer, EMT is associated with tumor development, invasion, metastasis, and treatment resistance 39. Our research revealed that MTSS1 participates in TGF‐β1‐induced EMT in glioma cells and miR-182 may work as an important biological target regulating MTSSI in proliferation and invasion of glioma cells.

Considering the downregulation of MTSS1 expression in glioma tissue and its biological function of inhibiting the proliferation and invasion of glioma cells, it is necessary to explore the molecular mechanism underlying MTSS1 downregulation. Currently, epigenetics is a research hotspot in tumor biology, and its role in tumor occurrence, progression and prognosis has been explored. The molecular mechanisms involved in epigenetics include DNA methylation, hydroxylation and carboxylation modifications 40; MiRNAs have been confirmed to participate in many physiological processes during organism development as well as in the pathophysiological processes of many diseases 41. Many research reports have shown that miRNAs can be classified as oncogenes or tumor suppressor miRNAs play a key role in the differentiation, proliferation, apoptosis, invasion and metastasis of tumor cells 42. In this study, we hypothesized the downregulation of MTSS1 expression in gliomas may be associated with miRNAs. Through bioinformatics analysis, we identified a conserved miR-182 binding site in the 3'-UTR of MTSS1 mRNA. Then we assessed the expression of miR-182 in glioma tissues and analyzed its relationship with MTSS1 expression. The expression of miR-182 was negatively correlated with the expression of MTSS1. Further experiments showed that after functional inhibition of miR-182 in glioma cells, the expression of MTSS1 was significantly upregulated. Assays with the double luciferase reporter gene system also confirmed that miR-182 can directly affect the expression of MTSS1. Moreover, functional inhibition of miR-182 suppressed the proliferation and invasion of glioma cells, similar to overexpression of MTSS1.

According to current reported research, the role of miR-182 in human tumors be two-sided—miR-182 may promote or inhibit cancers in a tissue-specific manner. The expression of miR-182 in high-grade serous ovarian cancer is significantly upregulated. Overexpression of miR-182 strongly promotes the malignant transformation of normal ovarian epithelial cells and enhances the invasion and metastasis abilities of ovarian cancer cells *in vitro* and *in vivo* 43. MiR-182 is also overexpressed in malignant melanoma cells and tissues; downregulation of miR-182 expression can inhibit the migration and invasion and promote the apoptosis of melanoma cells, and miR-182 plays a biological role by targeting FoxO_3_ for inhibition in melanoma 44. Recently, the overexpression of miR-182 was reported to be closely related to the clinical characteristics and poor prognosis of colorectal cancer 45, 46, and the abundance of miR-182 in the circulating peripheral blood can be used as a molecular marker to evaluate the progression of colorectal cancer 47. However, miR-182 is downregulated in gastric adenocarcinoma cells. Overexpression of miR-182 can inhibit the proliferation of gastric adenocarcinoma cells by targeting the expression of CREB1 48. The expression of miR-182 is downregulated in lung cancer and is correlated with upregulated expression of RGS17. Overexpression of miR-182 can inhibit the proliferation and anchorage-independent growth of lung cancer cells by downregulating the expression of RGS17 49. The role of miR-182 in human tumors remains to be further studied.

The results of this study preliminarily suggested the expression pattern and possible biological function of the miR-182/MTSS1 signaling axis in glioma. Therefore, our study laid a solid foundation for the diagnosis and treatment of glioma through understanding the function and mechanisms of MTSS1 in glioma. However, the function and molecular mechanism still need to be further confirmed by *in vitro* and *in vivo* experiments.

## Supplementary Material

Supplementary figures and tables.

## Figures and Tables

**Table 1:** Correlations between MTSS1 levels and clinicopathological parameters in glioma patients

Factors	Numbers	Mean ± SD	*P*-value
**Gender**			0.347
Male	26	0.29±0.26	
Female	8	0.26±0.37	
**Age**			0.771
<50	7	0.34±0.31	
≥50	27	0.26±0.25	
**Smoking history**			0.354
No	19	0.33±0.31	
Yes	15	0.22±0.19	
**Alcohol consumption**			0.817
No	22	0.31±0.30	
Yes	12	0.23±0.18	
**T stage**			0.0005***
T1+T2	13	0.47±0.26	
T3+T4	21	0.16±0.19	
**Clinical stage**			0.001***
I+II	11	0.48±0.28	
III+IV	23	0.18±0.19	

*P<0.05, ***P<0.001, *****P<0.0001.

## Abbreviations

MTSS1: metastasis suppressor protein
EMT: epithelial-mesenchymal transition
miRNAs: microRNAs
MIM: missing in metastasis
TGF-β1: transforming growth factor- beta 1
DMEM: dulbecco's modified eagle's medium
NC: negative control
3'-UTR: 3'-untranslated region
ANOVA: analysis of variance
IMD: IRSp53 and MIM Domain
lncRNA: long noncoding RNA
CREB1: CAMP responsive element binding protein 1

## References

[1] Balss J, Meyer J, Mueller W, Korshunov A, Hartmann C, von Deimling A (2008). Analysis of the IDH1 codon 132 mutation in brain tumors. Acta Neuropathol.

[2] Beier CP, Kumar P, Meyer K, Leukel P, Bruttel V, Aschenbrenner I (2012). The cancer stem cell subtype determines immune infiltration of glioblastoma. Stem Cells Dev.

[3] Beiko J, Suki D, Hess KR, Fox BD, Cheung V, Cabral M (2014). IDH1 mutant malignant astrocytomas are more amenable to surgical resection and have a survival benefit associated with maximal surgical resection. Neuro Oncol.

[4] Alcantara Llaguno S, Chen J, Kwon CH, Jackson EL, Li Y, Burns DK (2009). Malignant astrocytomas originate from neural stem/progenitor cells in a somatic tumor suppressor mouse model. Cancer Cell.

[5] Bettegowda C, Agrawal N, Jiao Y, Sausen M, Wood LD, Hruban RH (2011). Mutations in CIC and FUBP1 contribute to human oligodendroglioma. Science.

[6] Bhat KPL, Balasubramaniyan V, Vaillant B, Ezhilarasan R, Hummelink K, Hollingsworth F (2013). Mesenchymal differentiation mediated by NF-kappaB promotes radiation resistance in glioblastoma. Cancer Cell.

[7] Houillier C, Wang X, Kaloshi G, Mokhtari K, Guillevin R, Laffaire J (2010). IDH1 or IDH2 mutations predict longer survival and response to temozolomide in low-grade gliomas. Neurology.

[8] Jiang H, Ren X, Cui X, Wang J, Jia W, Zhou Z (2013). 1p/19q codeletion and IDH1/2 mutation identified a subtype of anaplastic oligoastrocytomas with prognosis as favorable as anaplastic oligodendrogliomas. Neuro Oncol.

[9] Jiao Y, Killela PJ, Reitman ZJ, Rasheed AB, Heaphy CM, de Wilde RF (2012). Frequent ATRX, CIC, FUBP1 and IDH1 mutations refine the classification of malignant gliomas. Oncotarget.

[10] Jin G, Reitman ZJ, Spasojevic I, Batinic-Haberle I, Yang J, Schmidt-Kittler O (2011). 2-hydroxyglutarate production, but not dominant negative function, is conferred by glioma-derived NADP-dependent isocitrate dehydrogenase mutations. PLoS ONE.

[11] Jones DTW, Kocialkowski S, Liu L, Pearson DM, Bäcklund LM, Ichimura K (2008). Tandem duplication producing a novel oncogenic BRAF fusion gene defines the majority of pilocytic astrocytomas. Cancer research.

[12] De Craene B, Berx G (2013). Regulatory networks defining EMT during cancer initiation and progression. Nat Rev Cancer.

[13] Huang L, Wu RL, Xu AM (2015). Epithelial-mesenchymal transition in gastric cancer. Am J Transl Res.

[14] Ambros V (2004). The functions of animal microRNAs. Nature.

[15] Bartel DP (2018). Metazoan MicroRNAs. Cell.

[16] Lu TX, Rothenberg ME (2018). MicroRNA. J Allergy Clin Immunol.

[17] Bartel DP (2004). MicroRNAs: genomics, biogenesis, mechanism, and function. Cell.

[18] Zealy RW, Wrenn SP, Davila S, Min KW, Yoon JH (2017). microRNA-binding proteins: specificity and function. Wiley Interdiscip Rev RNA.

[19] Wang D, Lu G, Shao Y, Xu D (2018). MiR-182 promotes prostate cancer progression through activating Wnt/beta-catenin signal pathway. Biomed Pharmacother.

[20] Chen G, Yu L, Dong H, Liu Z, Sun Y (2019). MiR-182 enhances radioresistance in non-small cell lung cancer cells by regulating FOXO3. Clin Exp Pharmacol Physiol.

[21] Zhang S, Li G, Liu C, Lu S, Jing Q, Chen X (2019). miR-30e-5p represses angiogenesis and metastasis by directly targeting AEG-1 in squamous cell carcinoma of the head and neck. Cancer Sci.

[22] Qin Y, Wang J, Zhu G, Li G, Tan H, Chen C (2019). CCL18 promotes the metastasis of squamous cell carcinoma of the head and neck through MTDH-NF-kappaB signalling pathway. J Cell Mol Med.

[23] Tan H, Zhu G, She L, Wei M, Wang Y, Pi L (2017). MiR-98 inhibits malignant progression via targeting MTDH in squamous cell carcinoma of the head and neck. Am J Cancer Res.

[24] Liu Y, Yu C, Qiu Y, Huang D, Zhou X, Zhang X (2012). Downregulation of EphA2 expression suppresses the growth and metastasis in squamous-cell carcinoma of the head and neck *in vitro* and *in vivo*. J Cancer Res Clin Oncol.

[25] Yu C, Liu Y, Tan H, Li G, Su Z, Ren S (2014). Metadherin regulates metastasis of squamous cell carcinoma of the head and neck via AKT signalling pathway-mediated epithelial-mesenchymal transition. Cancer Lett.

[26] Christofori G (2006). New signals from the invasive front. Nature.

[27] Wang H, Zhao Y, Cao L, Zhang J, Wang Y, Xu M (2019). Metastasis suppressor protein 1 regulated by PTEN suppresses invasion, migration, and EMT of gastric carcinoma by inactivating PI3K/AKT signaling. J Cell Biochem.

[28] Malta TM, de Souza CF, Sabedot TS, Silva TC, Mosella MS, Kalkanis SN (2018). Glioma CpG island methylator phenotype (G-CIMP): biological and clinical implications. Neuro-oncology.

[29] Vadakekolathu J, Al-Juboori SIK, Johnson C, Schneider A, Buczek ME, Di Biase A (2018). MTSS1 and SCAMP1 cooperate to prevent invasion in breast cancer. Cell Death Dis.

[30] Kawabata Galbraith K, Fujishima K, Mizuno H, Lee S-J, Uemura T, Sakimura K (2018). MTSS1 Regulation of Actin-Nucleating Formin DAAM1 in Dendritic Filopodia Determines Final Dendritic Configuration of Purkinje Cells. Cell Rep.

[31] Machesky LM, Johnston SA (2007). MIM: a multifunctional scaffold protein. J Mol Med.

[32] Dawson JC, Bruche S, Spence HJ, Braga VMM, Machesky LM (2012). Mtss1 promotes cell-cell junction assembly and stability through the small GTPase Rac1. PLoS ONE.

[33] Luxen D, Gielen GH, Waha A, Isselstein L, Müller T, Koch P (2017). MTSS1 is epigenetically regulated in glioma cells and inhibits glioma cell motility. Transl Oncol.

[34] Giacobbe A, Compagnone M, Bongiorno-Borbone L, Antonov A, Markert EK, Zhou JH (2016). p63 controls cell migration and invasion by transcriptional regulation of MTSS1. Oncogene.

[35] Liu K, Jiao X-D, Hao J-L, Qin B-D, Wu Y, Chen W (2019). MTSS1 inhibits metastatic potential and induces G2/M phase cell cycle arrest in gastric cancer. Onco Targets Ther.

[36] Du P, Wang S, Tang X, An C, Yang Y, Jiang WG (2017). Reduced Expression of Metastasis Suppressor-1 (MTSS1) Accelerates Progression of Human Bladder Uroepithelium Cell Carcinoma. Anticancer Res.

[37] Condeelis J, Pollard JW (2006). Macrophages: obligate partners for tumor cell migration, invasion, and metastasis. Cell.

[38] Brown AS, Meera P, Altindag B, Chopra R, Perkins EM, Paul S (2018). MTSS1/Src family kinase dysregulation underlies multiple inherited ataxias. Proc Natl Acad Sci USA.

[39] Pastushenko I, Blanpain C (2019). EMT Transition States during Tumor Progression and Metastasis. Trends Cell Biol.

[40] Leu S, von Felten S, Frank S, Vassella E, Vajtai I, Taylor E (2013). IDH/MGMT-driven molecular classification of low-grade glioma is a strong predictor for long-term survival. Neuro Oncol.

[41] Liu C, Sage JC, Miller MR, Verhaak RG, Hippenmeyer S, Vogel H (2011). Mosaic analysis with double markers reveals tumor cell of origin in glioma. Cell.

[42] Louis DN, Ohgaki H, Wiestler OD, Cavenee WK, Burger PC, Jouvet A (2007). The 2007 WHO classification of tumours of the central nervous system. Acta Neuropathol.

[43] Lyden D, Hattori K, Dias S, Costa C, Blaikie P, Butros L (2001). Impaired recruitment of bone-marrow-derived endothelial and hematopoietic precursor cells blocks tumor angiogenesis and growth. Nat Med.

[44] Malmstrom A, Gronberg BH, Marosi C, Stupp R, Frappaz D, Schultz H (2012). Temozolomide versus standard 6-week radiotherapy versus hypofractionated radiotherapy in patients older than 60 years with glioblastoma: the Nordic randomised, phase 3 trial. Lancet Oncol.

[45] Mentlein R, Hattermann K, Held-Feindt J (2012). Lost in disruption: role of proteases in glioma invasion and progression. Biochim Biophys Acta.

[46] Nakano I, Dougherty JD, Kim K, Klement I, Geschwind DH, Kornblum HI (2007). Phosphoserine phosphatase is expressed in the neural stem cell niche and regulates neural stem and progenitor cell proliferation. Stem Cells.

[47] Noushmehr H, Weisenberger DJ, Diefes K, Phillips HS, Pujara K, Berman BP (2010). Identification of a CpG island methylator phenotype that defines a distinct subgroup of glioma. Cancer Cell.

[48] Ohgaki H, Dessen P, Jourde B, Horstmann S, Nishikawa T, Di Patre PL (2004). Genetic pathways to glioblastoma: a population-based study. Cancer Res.

[49] Parker M, Mohankumar KM, Punchihewa C, Weinlich R, Dalton JD, Li Y (2014). C11orf95-RELA fusions drive oncogenic NF-kappaB signalling in ependymoma. Nature.

